# Effect of Cu Ion Concentration on Microstructures and Mechanical Properties of Nanotwinned Cu Foils Fabricated by Rotary Electroplating

**DOI:** 10.3390/nano11082135

**Published:** 2021-08-22

**Authors:** Yu-Wen Hung, Dinh-Phuc Tran, Chih Chen

**Affiliations:** 1 Department of Materials Science and Engineering, National Yang Ming Chiao Tung University, Hsinchu 30010, Taiwan; patty8862000@gmail.com (Y.-W.H.); trandinhphuc1508@gmail.com (D.-P.T.); 2 Department of Materials Science and Engineering, National Chiao Tung University, Hsinchu 30010, Taiwan

**Keywords:** rotary electrodeposition, nanotwinned Cu, concentration of Cu ions, columnar grain structures, mechanical properties

## Abstract

Rotary electroplating was employed to fabricate high-strength nanotwinned copper (nt-Cu) foils serving as a current collector for high energy-density lithium ion batteries (LIBs). The effect of Cu ion concentration on the microstructural and mechanical properties of the nt-Cu foils was then investigated. Formation of nano-scaled grains was found at the bottom. Its size gradually increases toward the top surface to form a microstructural mixture of gradient nano-scaled and columnar grains in the upper region. Experimental results show that the grains and elongation of the nt-Cu foils increase with increasing concentration of Cu ions. However, a trade-off between tensile strength and elongation is present. The elongation of nt-Cu foils has been enhanced by 22% (from 3.1% to 3.8%) while 8.3% and 3.9% reductions in ultimate tensile strength (UTS) and yield stress (YS) are seen. The current study shows a promising method to tune and optimize the microstructure and mechanical properties of such nt-Cu foils for various applications.

## 1. Introduction

Recently, renewable energy demand for the advanced electric vehicle industry has significantly increased. This has led to a mass desire for lithium-ion batteries (LIBs) [1,2,3,4,5,6,7]. In general, the anode current collector of an LIB is made of several copper (Cu) foils coated with an active material [6,7,8,9,10,11]. In order to increase the energy density, the weight and volume of those Cu foils must be reduced. However, during a charge-discharge process, various chemical reactions occur in LIBs resulting in a volume expansion. It may lead to significant stresses in those Cu foils and eventually damage LIBs. Thus, the mechanical properties of the Cu foils should be considered [6,7,8,9,10,11,12].

The main strengthening mechanisms of metals are grain size strengthening, work hardening, precipitation hardening, and solid solution strengthening. These can enhance Cu foil strength, but may lead to an increase in electrical resistance [13]. It has been demonstrated that an equiaxial nanotwinned (nt) structure significantly improves the mechanical strength [14,15,16] while its conductivity remains unchanged [17] because it does not cause electron scattering [18,19,20,21,22]. Therefore, such high-strength nt-Cu foils have potential for use as an LIB anode current collector. They could be fabricated using a traditional rolling process. However, formation of variously large defects and high impurity of Cu are inevitable. Additionally, most of the previous studies focused on the influences of current density and electroplating temperature [23,24,25,26]. To date, the effect of Cu ion concentration on the microstructure and mechanical properties of nt-Cu foils has not been fully investigated.

In this study, high-strength nt-Cu foils with gradient nanograined structure were fabricated by rotary electroplating. The rotary electrodeposition method is highly advantageous thanks to its high deposition rate, high tensile strength of films, compatibility for large foils, and the easiness to control the foil thickness and defects. Therefore, the fabrication cost of such a rotary electroplating technique would be greatly reduced. Thus, it has potential for the mass production of high tensile strength nt-Cu foils. The effect of concentration of Cu ions on the microstructure and crystal orientation is then characterized. Additionally, its influence on the mechanical properties of the nt-Cu foils is further discussed. Experimental results are also correlated with theoretical calculation using Hall-Petch equation to analyze the combined strengthening mechanism of columnar nanotwin and nano-grained gradient.

## 2. Experimental

**Figure 1:**
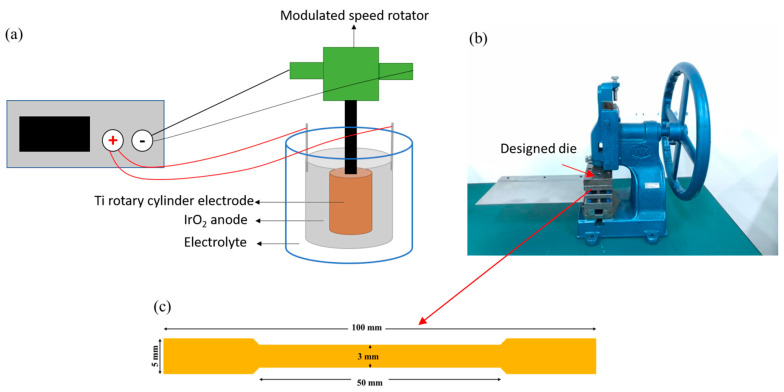
(**a**) Schematic of the rotary system. (**b**) The punching machine and (**c**) detailed dimensions of the nt-Cu dog-bone-shaped samples.

In this study, rotary electroplating was applied to fabricate the nt-Cu foils, as shown in Figure 1a. The titanium (Ti) rotary cylinder cathode was connected to a modulated speed rotator (MSR). It was surrounded by an iridium dioxide (IrO_2_) coated anode. Those electrodes were submerged in a CuSO_4_ electrolyte. The cylinder cathode rotated continuously to provide a uniform electric flow. A constant rotation speed of 800 rpm was set during the electroplating. The electrolyte solution was composed of 100 g of H_2_SO_4_, 0.1 mL of HCl, and 20 mL of an organic additive dissolved in 1 L of deionized (DI) water. The Cu foils were deposited using different concentrations of Cu ions (Cu^2+^) of 0.3 M, 0.5 M, 0.8 M. We tuned the Cu ion concentration by adjusting the CuSO_4_ portion in the electrolyte. A current density of 15 ASD (A/dm^2^) was set, and various thicknesses (5, 10, and 15 μm) of nt-Cu foils were then electroplated. The resistivity (~2.81 µΩ.cm) of the nt-Cu foils was also measured, and it was comparable with that of regular Cu (~1.85 µΩ.cm).

The mechanical properties of the nt-Cu samples deposited with different concentrations of Cu^2+^ were analyzed by tensile tests (AGS-X, Shimadzu, Japan). The tensile tests were conducted with a constant strain rate of 4.17 × 10^−3^ 1/s at room temperature. In this study, the nt-Cu foils were electroplated on a titanium (Ti) rotary cylinder cathode and the adhesion of the Cu foils to the Ti rotary cylinder was weak. Therefore, it was very easy to peel them off without damaging the nt-Cu foils. A designed die was used to cut the nt-Cu foils into various dog-bone shaped samples using a punching machine (Figure 1b). The thickness of each dog bone nt-Cu sample was carefully measured by examining its weight. To minimize the measurement error and to ensure the data repeatability, five dog bone shaped specimens (Figure 1c) were cut from each as-deposited Cu foil. The ultimate tensile strength (UTS), yield strength (YS), and elongation were obtained from five samples for each electroplating parameter. Besides, the extreme values were removed and the average was chosen as the typical characteristic. The microstructures of the nt-Cu foils were characterized using a focused ion beam system (FIB, Helios G3CX, FEI, Hillsboro, Oregon, USA) and a transmission electron microscope (TEM, TALOS-F2OOX, FEI, Hillsboro, Oregon, USA). Grain size and orientation of the nt-Cu foils were analyzed using electron backscattered diffraction (EBSD, Oxford instruments, Abingdon, United Kingdom) and X-ray diffraction (XRD, BRUKER D2 PHASER, Karlsruhe, Germany).

## 3. Results and Discussion

### 3.1. Effect of Cu^2+^ Concentration on Microstructure and Grain Size

**Figure 2:**
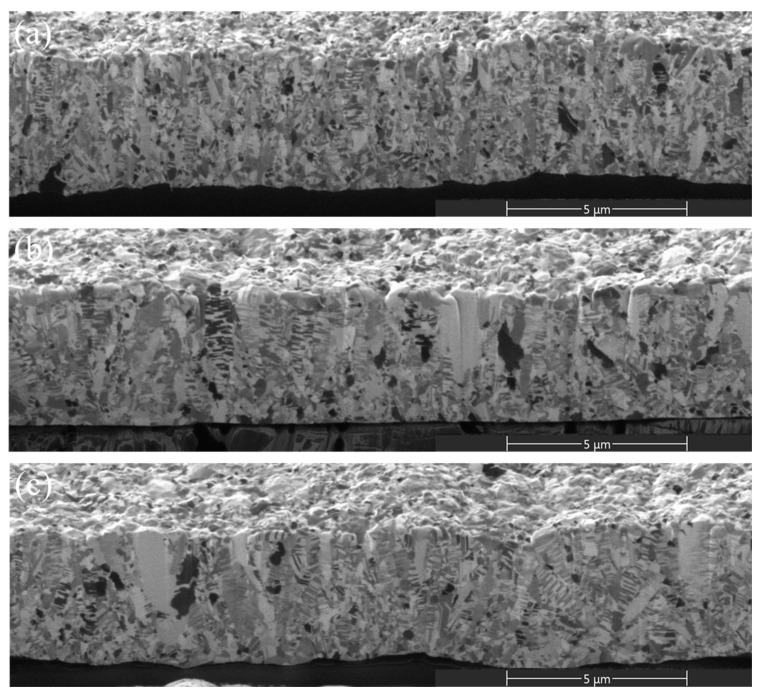
Cross-sectional FIB micrographs of the 5-μm thick nt-Cu foils electroplated with different concentrations of Cu^2+^: (**a**) 0.3 M; (**b**) 0.5 M; (**c**) 0.8 M.

**Figure 3:**
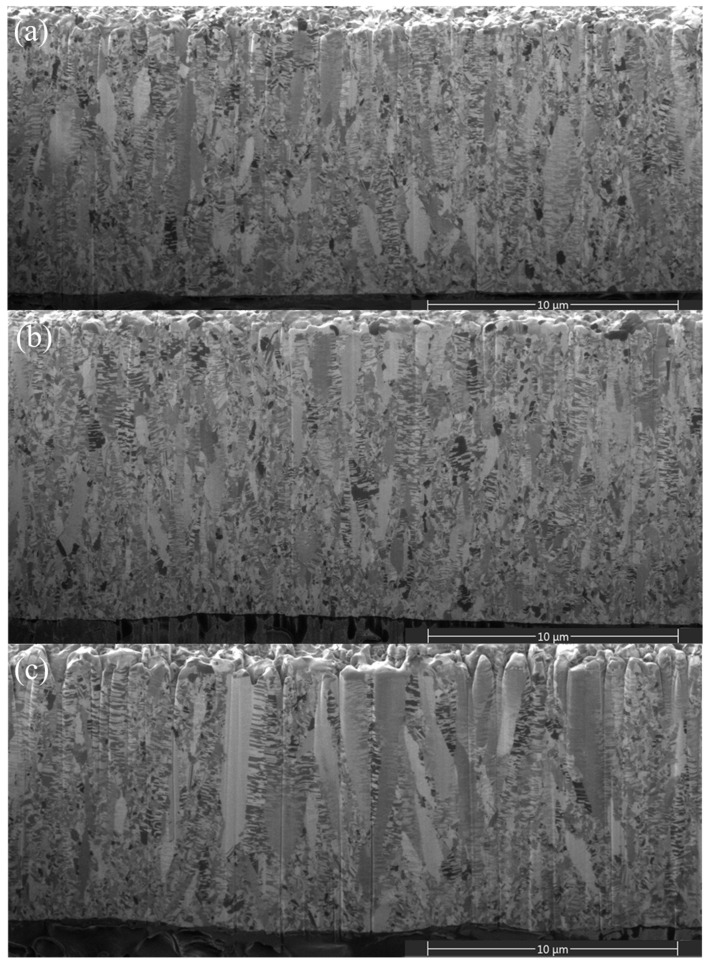
Cross-sectional FIB micrographs of the 15-μm thick nt-Cu foils electroplated with different concentrations of Cu^2+^: (**a**) 0.3 M; (**b**) 0.5 M; (**c**) 0.8 M.

**Figure 4:**
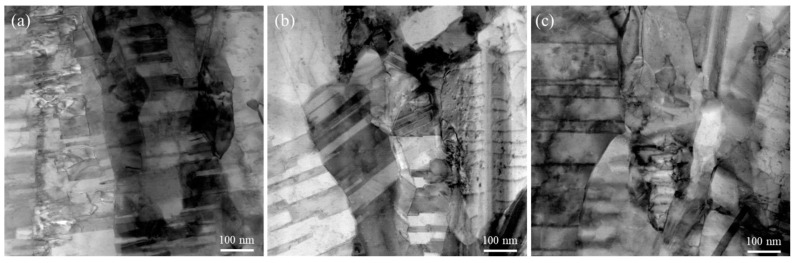
TEM micrographs of the 15-μm thick nt-Cu foils electroplated with different concentrations of Cu^2+^: (**a**) 0.3 M; (**b**) 0.5 M; (**c**) 0.8 M.

Cross-sectional micrographs of the 5-μm and 15-μm thick nt-Cu foils electroplated with different concentrations of Cu^2+^ are shown in Figure 2 and Figure 3. We found that grains are smaller at the bottom and gradually increase toward the top surface (Figure 2). As the thickness becomes larger, the columnar grains form and extend. Such a gradient nanostructure is more obvious in the 15-μm nt-Cu foils (Figure 3). With a low concentration of Cu^2+^ (0.3 M), randomly nano-scaled grains dominate (Figure 3a), while columnar grains prevail in the foils electroplated with a greater Cu^2+^ concentration (Figure 3c). We found that the Cu^2+^ concentration also affects the twin density of the electroplated nt-Cu foils. The twin spacing of the nt-Cu foils electroplated with a 0.3-M Cu^2+^ is ~55 nm (Figure 3a). It widens to 62 nm as electroplated with a 0.5-M Cu^2+^ (Figure 3b). When the Cu^2+^ concentration further increased to 0.8 M, a twin spacing of 71 nm was found (Figure 3c). We also performed TEM analysis for more accurate characterization of their twin spacing and microstructures. As shown in Figure 4, the twin spacing of the 15- μm thick foils electroplated with 0.3-, 0.5-, and 0.8-M Cu^2+^ is 57, 63, and 73 nm, respectively. These are comparable with the above FIB results. We can conclude that the twin spacing decreases with a reduction in Cu^2+^ concentration.

**Figure 5:**
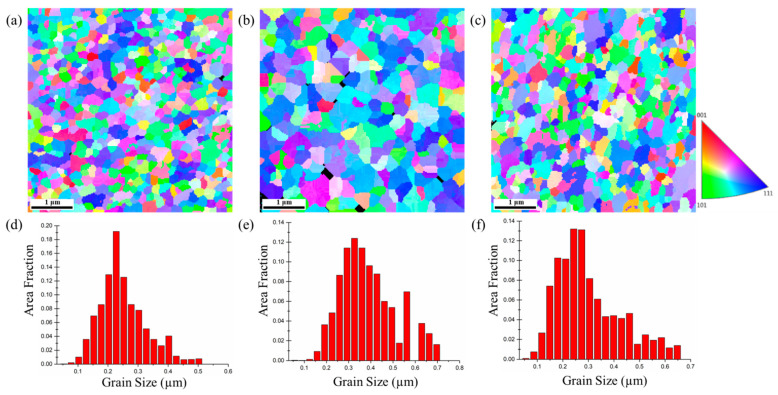
Plan-view EBSD micrographs and grain size distributions of the 5-μm thick nt-Cu foils electroplated with different concentrations of Cu^2+^: (**a**,**d**) 0.3 M; (**b**,**e**) 0.5 M; (**c**,**f**) 0.8 M, respectively.

**Figure 6:**
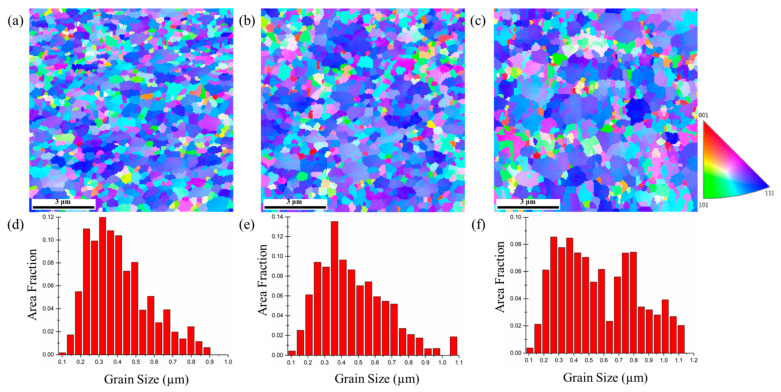
Plan-view EBSD micrographs and grain size distributions of the 10-μm thick nt-Cu foils electroplated with different concentrations of Cu^2+^: (**a**,**d**) 0.3 M; (**b**,**e**) 0.5 M; (**c**,**f**) 0.8 M, respectively.

**Figure 7:**
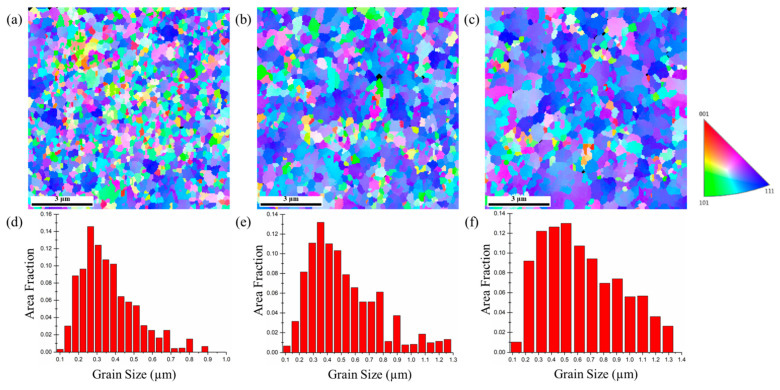
Plan-view EBSD micrographs and grain size distributions of the 15-μm thick nt-Cu foils electroplated with different concentrations of Cu^2+^: (**a**,**d**) 0.3 M; (**b**,**e**) 0.5 M; (**c**,**f**) 0.8 M, respectively.

The plane-view EBSD micrographs of the 5-μm, 10-μm, and 15-μm thick nt-Cu foils are shown in Figure 5, Figure 6 and Figure 7. The measured grain size is listed in Table 1. We found that the grain size of the 5-μm thick nt-Cu electroplated with different Cu^2+^ concentrations (Figure 5) is nearly identical. Its grain size is averaged as 0.21 μm. The average grain size of the 10-μm thick foils electroplated with 0.3, 0.5, and 0.8 M of Cu^2+^ is 0.24, 0.32, and 0.33 μm, respectively (Figure 6). Note that the blue regions are employed to represent the <111> orientation. We found that most grains in the 10-μm nt-Cu are <111>-orientated. For the thicker foils (15-μm), its average grain size is 0.26, 0.33, and 0.37 μm, respectively, and the <111>-orientated microstructures are also observed (Figure 7). With a high Cu ion concentration, the Cu atoms scatter densely. At the initial stage of electroplating, a high concentration of Cu ions results in a decrease in nuclei population density produced on the substrate [27]. The Cu atoms travel toward to minimize the surface energy during electrodeposition. They merge with the nearest neighbors and form nuclei. A smaller proximity of atoms (high Cu ion concentration) will thus lead to the grouping of a larger nucleus. Generally, grain size increases with increasing electroplated foil thickness and Cu^2+^ concentration. Over potential (*η*) would increase as the copper ion concentration reduces [28,29]. The larger the over potential (*η*), the easier it is to obtain fine grains. In other words, a high Cu ion concentration would result in the columnar grains. The correlation of grain size and foil thickness measured using EBSD provides more evidence of the gradient nanostructure. The results are consistent with the aforementioned FIB analysis.

### 3.2. Effect of Cu^2+^ Concentration on Crystal Orientation

**Figure 8:**
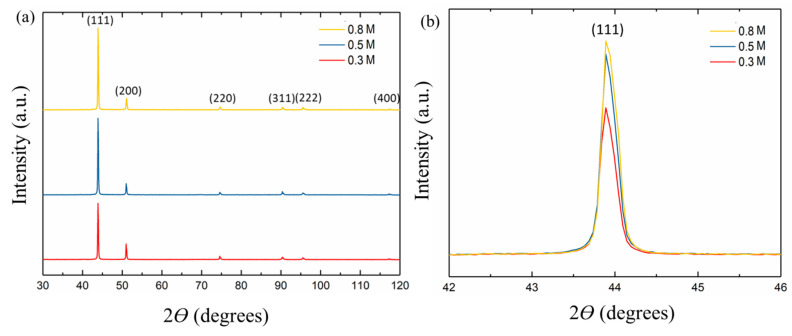
(**a**) XRD patterns and (**b**) (111) intensities of the 15-μm thick nt-Cu foils electroplated with different Cu^2+^ concentrations.

**Figure 9:**
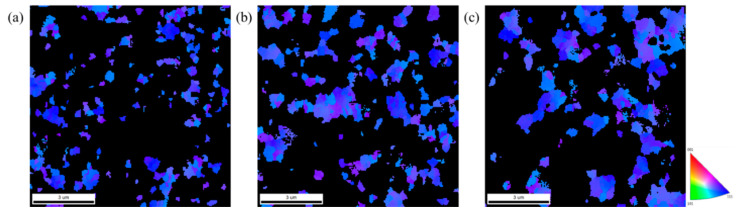
Plan-view EBSD (111) orientation maps (OIMs) of the 15-μm thick nt-Cu foils electroplated with different concentrations of Cu^2+^: (**a**) 0.3 M; (**b**) 0.5 M; (**c**) 0.8 M.

The XRD patterns of the 15-μm nt-Cu foils electroplated with various Cu^2+^ concentrations are shown in Figure 8. It can be seen that the intensity of (111) peaks increases with an increase in Cu^2+^ concentration (Figure 8b). In this study, we attached the nt-Cu samples on a Si substrate to measure their XRD patterns and set the Si intensity as a reference. We then removed the Si signal to obtain the XRD patterns with a focus on the Cu orientation. The ratio of (111)/(200) peak intensity is 3.43, 6.29, and 6.66 as electroplated with 0.3-, 0.5-, and 0.8-M Cu^2+^, respectively. We have precisely calculated the ratio of (111)-oriented grains with the corresponding concentration of Cu ions using EBSD. Note that the precision of EBSD measurements is greater than the XRD counterparts. The XRD patterns were employed in this study to further support the EBSD results. The plan-view EBSD (111) orientation maps of the 15-μm nt-Cu foils are shown in Figure 9. The ratio of (111)-oriented grains is 18.4%, 25.5%, and 27.0% as electroplated with 0.3-, 0.5-, and 0.8-M Cu^2+^, respectively. The results confirm that the nt-Cu foil deposited with a higher Cu^2+^ concentration possesses a greater (111)-preferred orientation. Note that a critical over potential (*η*) can form the field-oriented texture (FT) type with a high degree of twinning perpendicular to the (111) growth direction. Over potential (*η*) would increase as the Cu ion concentration reduces [28,29]. The low Cu ion concentration results in unoriented dispersion (UD) because the over potential (*η*) is above a critical value, which causes less preferred orientation (111) compared to that with a higher counterpart.

### 3.3. Mechanical Properties

**Figure 10:**
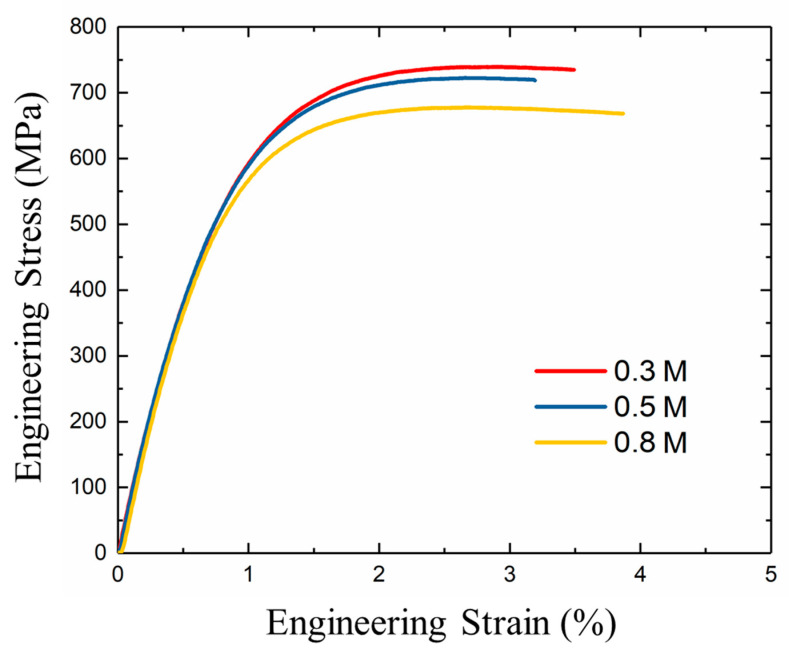
Stress-strain characteristics of the 15-μm thick nt-Cu foils electroplated with different concentrations of Cu^2+^.

The typical tensile engineering stress–strain relationships of samples electroplated with different Cu^2+^ concentrations are shown in Figure 10. The detailed data of mechanical properties are summarized in Table 2. The UTS of samples deposited with 0.3, 0.5, and 0.8 M of Cu^2+^ is 739.8 MPa, 720.1 MPa, and 678.5 MPa, respectively. The UTS of Cu foils generally increases with decreasing in Cu^2+^ concentration (Figure 10), and the corresponding elongation is 3.4%, 3.1%, and 3.8%, respectively. We discovered a trade-off between tensile strength and elongation of those nt-Cu foils. The elongation of the nt-Cu foils has been enhanced by 22% (from 3.1% to 3.8%) while the ultimate tensile strength (UTS) and yield stress (YS) are slightly decreased by 8.3% and 3.9%, respectively. Metals with gradient structures have been demonstrated to possess excellent tensile properties due to the strain gradient generation and incompatible plastic deformation along the gradient depth under tension [30,31]. The primary strengthening mechanism of the microstructural mixture of gradient nanoscaled and columnar nt grains can be attributed to the dislocation pile-ups in grain interior and twin boundaries during deformation. They are considered as strong barriers to suppress the dislocation movement and strain localization at grain and twin boundaries.

As aforementioned, columnar grains become straighter and larger with increasing in Cu^2+^ concentration (Figure 3, Figure 4, Figure 7 and Figure 8). Thus, UTS and YS are accordingly reduced. Moreover, the (111) orientation is more intensive as the concentration of Cu^2+^ increases. Note that a FCC structure has three slip directions on the (111)-closed-packed plane. Under deformation, the activation of multiple slipping systems could benefit the smooth sliding of grains and twin boundaries and suppress the stress concentration in the nt-Cu foils. Thus, an enhanced elongation can be obtained with a greater (111) orientation ratio.

**Figure 11:**
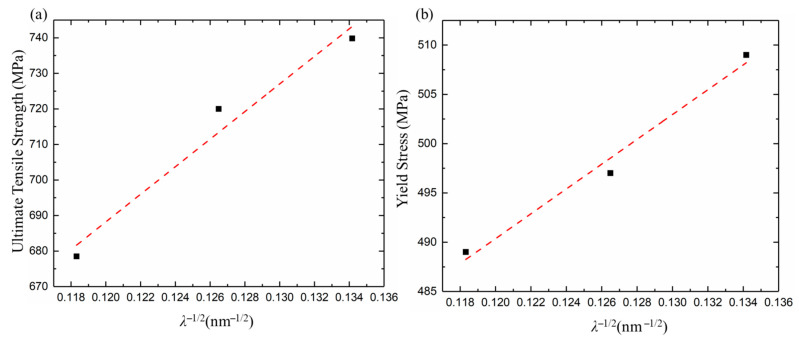
(**a**) UTS and (**b**) YS versus $\lambda^{-1/2}$ scattering plots using Equation (2).

It is noted that the movement of dislocations is blocked by the grain boundaries. The finer grains would result in lower ductility due to the fact that the dislocation slip is prohibited by denser grain boundaries. Additionally, a low Cu^2+^ concentration causes an increase in twin density resulting in further blocking of dislocation movement. Thus, the elongation decreases with a reduction of Cu^2+^ concentration.With the premise that the grain size must be larger than 30 nm, and the temperature must be below an equicohesive temperature (ECT), YS can be estimated using a classical Hall-Petch equation [32,33,34,35] as follows,

(1) $$\sigma_{y}=\sigma_{0}+\frac{K_{y}}{\sqrt{d}}$$

where $\sigma_{0}$ is the friction stress of Cu; $k_{y}$ is the strengthening coefficient, and *d* is the average grain diameter [24]. However, the experimental value of YS is greater than the calculated value. The reason is that the classical Hall-Petch equation only considers the size of crystal grains. It does not account for the twin boundaries. It has been demonstrated that incorporating nanotwins in a certain material could significantly improve its mechanical properties [17]. The correlation between twin spacing and UTS or YS can be characterized by the below Hall-Petch equation [22,25]:

(2) $$\sigma_{y}=\sigma_{0}+\frac{k}{\sqrt{\lambda }}$$

where $\sigma_{0}$ is the friction stress of Cu (25 MPa); *λ* is the average twin spacing; and *k* is the strengthening coefficient. The plot of UTS as a function of $\lambda^{\frac{-1}{2}}$ is shown in Figure 11a. The corresponding strengthening (*k*) and determination (*R*^2^) coefficients are calculated as 3880.3 MPa nm^1/2^ and 0.9666, respectively. YS as a function of $\lambda^{\frac{-1}{2}}$ is also plotted in Figure 11b. The *k* and *R*^2^ coefficients are estimated as 1258.8 MPa nm^1/2^ and 0.9825, respectively. The results show that UTS and YS increase with a decrease in twin spacing. A greater ductility of the nt-Cu can be fabricated with a higher concentration of Cu^2+^. In fact, the Hall-Petch is not the primary strengthening mechanism. In order to further explore the contribution of twin spacing to YS, the correlation between twin spacing and YS can be characterized by the confined layer slip (CLS) model [21,24,36,37],

(3) $$\sigma_{\mathrm{cls}}=M\beta \frac{\mu b}{\lambda }\ln\frac{\alpha \lambda }{b}$$

where *M* is the Taylor factor, the Schmid factor (0.45), *β* is Poisson’s ratio (0.218), *α* is the material constant (0.16), *μ* is shear modulus (46 GPa), and *b* is the Burger’s vector component (0.256 nm). Substituting the twin spacing into Equation (3), we obtained the YS of 357.6, 332.6, and 298.6 MPa corresponding with the 0.3-, 0.5-, and 0.8-M Cu^2+^, respectively. In this study, we fabricated the nt-Cu foils with a microstructural mixture of nanotwinned and gradient grains. The anisotropic deformation behavior of the nt-Cu foils results in the activation of multiple dislocation slip systems, where twin boundaries benefit from the highest strengthening effect of gradient and nanotwinned grains. Thus, it leads to excellent tensile strength and ductility of the nt-Cu foils.

## 4. Conclusions

In this study, we successfully fabricated high-strength nt-Cu foils with columnar gradient grains by direct current rotary electroplating. The bottom of nt-Cu foils consists of nano-scaled grains, and the microstructure of the upper region is a mixture of gradient nano-scaled and columnar grains. This results in great tensile strength and high ductility of our electroplated nt-Cu foils. The foils deposited with a lower concentration of Cu^2+^ possess finer grains and a lower ratio of (111)-preferred orientation. As the concentration of Cu^2+^ increases, the crystal grain size becomes larger and a mixture of gradient nano-scaled and columnar grains is found. In addition, the ratio of (111) crystal orientation becomes more obvious with increasing concentration of Cu^2+^. Thus, the difference in microstructure is reflected in the mechanical properties. The elongation was enhanced by 22.6% as the Cu^2+^ concentration increased to 0.8 M. However, its corresponding YS and UTS slightly decreased by 3.9% and 8.3%. We found that the columnar nt structure with high (111)-preferred orientation can notably improve ductility with slightly reduced mechanical strength. The findings are hoped to shed light on potential use of such great tensile strength and high ductility nt-Cu foils for advanced LIBs.

## Figures and Tables

**Table 1:** Grain size of the nt-Cu foils obtained using plan-view EBSD images.

Sample	5 μm	10 μm	15 μm
[Cu^2+^] = 0.3 M	0.21 μm	0.24 μm	0.26 μm
[Cu^2+^] = 0.5 M	0.21 μm	0.32 μm	0.33 μm
[Cu^2+^] = 0.8 M	0.21 μm	0.33 μm	0.37 μm

**Table 2:** Summary of the mechanical properties of the 15-μm thick nt-Cu foils electroplated with different concentrations of Cu^2+^. The calculated yield stress (YS) was obtained using the classical Hall-Petch equation (Equation (1)).

Sample	Yield Stress (MPa)	Calculated Yield Stress (MPa)	Ultimate Tensile Strength (MPa)	Fracture Elongation (%)
[Cu^2+^] = 0.3 M	509.9 ± 5.6	260.3	739.8 ± 10.5	3.4 ± 0.3
[Cu^2+^] = 0.5 M	497.7 ± 6.1	233.9	720.1 ± 8.2	3.1 ± 0.4
[Cu^2+^] = 0.8 M	489.8 ± 6.6	222.3	678.5 ± 7.4	3.8 ± 0.8

## Data Availability

The raw/processed data required to reproduce these findings cannot be shared at this time as the data also forms part of an ongoing study.
